# Changing the incentives for development of low-cost high-impact treatments

**DOI:** 10.1017/cts.2017.299

**Published:** 2018-02-02

**Authors:** Harry P. Selker, Herbert Pardes

**Affiliations:** 1 Tufts Clinical and Translational Science Institute, Tufts University; 2 Institute for Clinical Research & Health Policy Studies, Tufts Medical Center; 3 NewYork–Presbyterian Hospital, New York, NY, USA

Medical researchers work to develop and disseminate medical breakthroughs. They have shared the concerns of many regarding recent escalations in drug prices. There are many causes for these escalations.

One basic cause is our healthcare system is built on free-enterprise and economic incentives/requirements in almost every component of healthcare. Drug pricing policy elicits many different opinions. While not addressing all the issues involved in policy considerations, it would seem advisable that the key players develop a dialog to deal with these considerable challenges. One example requiring attention is the need for incentives for development of low-cost high-impact treatments.

Currently in the United States for medical treatment to get to market, aside from necessary scientific work and regulatory approvals, sufficient potential for profit must be apparent to justify an investment by a pharmaceutical company to undertake drug development, including clinical trials and marketing. For drugs not patented and/or in the public domain, there is little market protection. Consequently such investment is unlikely.

There are exceptions created by regulation, for example, “orphan” drugs. Manufacturers can obtain market protection that allow prices sufficient to generate profits that justify their investment.

The intent is to make available to patients medications that otherwise that might not be developed or marketed. But price escalations for medications with such protection have raised concerns about the desirability and/or long-term feasibility of this approach. Also, this mechanism is not applicable to “non-orphan” drugs for common diseases. Consequently, and paradoxically, nonpatented medications that may have the greatest impact on patients’ health and the public are least likely to market in that they attract no investment because of the absence of apparent profitability.

One of us has an example of this in his own research on the use of intravenous glucose-insulin-potassium (GIK) for acute coronary syndromes (ACS). This treatment has substantial potential to save lives and reduce damage from acute myocardial infarction (AMI, the consequence of unchecked ACS) [1]. A randomized placebo-controlled immediate clinical trial showed that GIK reduces the composite of cardiac arrest or mortality from ACS by 50%, and by 60% for ST segment elevation AMIs, the most severe type of AMI [2]. It also reduced AMI size by 80%, which may hold promise for a reduction in long-term heart failure. Understandably, given the dramatic nature of these results and their enhanced value by contrast to earlier different trials, the FDA requires a confirmatory trial. However, despite this trial’s results being very favorable for the largest cause of morbidity and mortality in the United States, the absence of a significant period of market exclusivity that would add to the return on investment (ROI) has deterred drug companies from supporting the confirmatory clinical trial and plans for marketing.

This situation is representative of an important gap in the drug development ecosystem and is adverse to both the interests of patients and the public’s health. In considering how this problem might be addressed, 3 paths might be considered: (1) creating directed incentives for pharmaceutical companies; (2) harnessing incentives that would leverage stakeholders across the healthcare ecosystem; and (3) creating an organization that could promote development of such treatments.

One solution for the lack of direct ROI on drugs for pharmaceutical companies such as GIK would be to develop incentives to encourage development of otherwise low ROI medicines. Many would claim there are potentially thousands of therapeutically valuable—and inexpensive—drug candidates sitting on lab and pharmaceutical company shelves that, if developed or repurposed, could reduce treatment costs and improve outcomes. Stronger incentives would be needed to encourage development of these products. Tools such as “priority review vouchers” have been effective in encouraging development of drugs for neglected and tropical diseases. And “market exclusively extension” has encouraged companies to test drugs in pediatric populations. Why not an incentive program (beyond a use patent) for low ROI drugs? Although other mechanisms would seem possible, a particularly attractive one would seem to be policy changes that made this change in the regulatory environment.

A second way to look at this is as an ecosystem problem. By only incenting drug manufacturers, we treat them as the only key player. In fact, key stakeholders also include patients, payers, employers, and the public—from economic, social, and health perspectives. Thus a way to leverage this broader ecosystem would have promise. This would require having incentives that engaged stakeholders in the healthcare value chain, both to take appropriate responsibility and to accrue benefits. Patients, payers, governmental agencies, the public, and policy-makers being included in system-wide incentives would presumably be important. Aside from policy changes, mechanisms for this might be a joint agreement among stakeholders to fund such projects, or a nonprofit or public benefit corporation that could pool resources.

A third approach would be engaging organizations with a public health focus, such as governments and foundations, to support the development of such treatments. This could be done in a focused effort, such as have been undertaken for HIV/AIDS and tropical diseases in developing countries. An argument might be that a nonprofit or public benefit company be created to develop the marketplace of such drugs. The criteria for supporting the development of a medication might include reduced expense, with a cost per quality-adjusted life year below some threshold, and that an important expected impact would be secured. Foundations, industry, and governments might all be interested. This would be made apparent most likely by discussions related to the second approach.

The 3 pathways outlined are just examples of approaches. There may be others, including ones of a global nature that would require overall healthcare system reform. The example used here is close at hand to one of our offices, but is certainly only one of many. The summary point is that we need to address the failure of the current marketplace to generate innovation for nonpatented inexpensive drugs for wide groups of patients. The needed conversation should include multiple stakeholders and review diverse models for addressing this challenge. Included should be patients and patient advocates, clinicians, payers (e.g., Medicare and Medicaid, private insurance companies, and employers), regulatory agencies, policy makers, pharmaceutical companies, academics and researchers, and others. We as clinical and translational researchers should consider this set of issues as part of our translational work in developing clinical research with a positive impact on the nation’s health. Our focus should be on fostering useful and valued medical breakthroughs of consequence to the entire public and contributing to the solution of dilemmas illustrated by the above.
